# Improving Neural Machine Translation by Filtering Synthetic Parallel Data

**DOI:** 10.3390/e21121213

**Published:** 2019-12-11

**Authors:** Guanghao Xu, Youngjoong Ko, Jungyun Seo

**Affiliations:** 1 Department of Engineering, Computer Science, Sogang University, Seoul 04107, Korea; guanghao412@gmail.com (G.X.); seojy@sogang.ac.kr (J.S.); 2 Applied Data Science, Sungkyunkwan University, Suwon 16419, Korea

**Keywords:** neural machine translation, back translation, bilingual word embeddings, synthetic data filtering

## Abstract

Synthetic data has been shown to be effective in training state-of-the-art neural machine translation (NMT) systems. Because the synthetic data is often generated by back-translating monolingual data from the target language into the source language, it potentially contains a lot of noise—weakly paired sentences or translation errors. In this paper, we propose a novel approach to filter this noise from synthetic data. For each sentence pair of the synthetic data, we compute a semantic similarity score using bilingual word embeddings. By selecting sentence pairs according to these scores, we obtain better synthetic parallel data. Experimental results on the IWSLT 2017 Korean→English translation task show that despite using much less data, our method outperforms the baseline NMT system with back-translation by up to 0.72 and 0.62 Bleu points for tst2016 and tst2017, respectively.

## 1. Introduction

Recent advances in neural machine translation (NMT) have achieved human parity on several language pairs given large-scale parallel corpora [1,2]. However, for many language pairs, the amount of parallel corpora is limited; this is a major challenge in building high-performance machine translation (MT) systems [3]. By contrast, there are plenty of monolingual data, which are easier to obtain.

Many approaches have been proposed to improve MT systems by leveraging monolingual data [4,5]. Sennrich et al. [6] proposed a back-translation approach to expand a parallel training corpus with synthetic parallel data. In this approach, the synthetic parallel data are constructed by translating the target-language monolingual data into the source language with a backward translation (target-to-source) model trained by a given parallel training corpus. Although this approach can generate a large amount of synthetic parallel data, there is no guarantee of its quality.

Regarding synthetic data filtering, Imankulova et al. [7] attempted to filter out those low-quality sentence pairs from the synthetic parallel data. To measure the quality of synthetic sentence pairs, they first translated synthetic source sentences to construct synthetic target sentences by using a forward translation (source-to-target) model. Then, for each sentence pair, the sentence-level Bleu [8] score between the target-language monolingual sentence and the target-language synthetic sentence was calculated. Finally, sentence pairs of the lower score were filtered out from the synthetic parallel corpus. By filtering out noisy sentence pairs, they obtained improvements over the baselines on several low-resourced translation tasks. However, they observed that translation performance did not improve when the size of monolingual data was large, i.e., over 1 million sentences. Furthermore, to calculate the sentence-level Bleu scores, they built an additional translation model to generate the target-language synthetic sentences.

Following the shared task on parallel corpus filtering introduced by Koehn et al. [9] at WMT2018 (Third Conference on Machine Translation), in this paper, we propose a simple and effective approach to filter out noisy sentence pairs from synthetic parallel data. Our approach is based on sentence-level cosine similarities of two sentence vectors, i.e., vector representations of the synthetic source sentence and the monolingual target sentence. We calculate the sentence vectors by averaging the word embeddings of each sentence. In addition, to locate the sentence vectors in a common vector space, we learn bilingual linear mappings between word embeddings of the source and the target language. The proposed method has two advantages: (1) no additional translation models are required to generate synthetic target sentences, and (2) semantic information of words in both synthetic and monolingual sentences are considered by using both source and target word embeddings. To the best of our knowledge, no previous works have investigated the similarity of the synthetic source and the target sentence in the context of synthetic parallel corpus filtering.

The remainder of this paper is structured as follows. In Section 2, we describe related research. In Section 3, we introduce our proposed filtering method. In Section 4, we present the experimental setup. In Section 5, we discuss the results of our experiment. Finally, in Section 6, we conclude the paper and suggest future work.

## 2. Related Work

Most of the methods of learning bilingual embeddings are supervised and rely on a small bilingual dictionary of a few thousand word pairs. Mikolov et al. [10] first proposed a cross-lingual embedding mapping method, which maps word embeddings in two languages by learning a linear transform. Xing et al. [11] found inconsistencies in the objective function of the linear transform, and proposed to constrain the linear transform as an orthogonal transform. Luong et al. [12] proposed a joint model that used both the context co-occurrence information through the monolingual component and the meaning equivalent signals from the bilingual constraint. They showed that the model was capable of learning bilingual representations of two languages, simultaneously preserving the monolingual clustering structures in each language. Artetxe et al. [13] proposed a framework of learning bilingual mappings of word embeddings, which generalized previous research.

Several studies examined the context of learning bilingual embeddings in a semi-supervised or unsupervised scenario, where the bilingual dictionary was much smaller. Artetxe et al. [14] proposed a self-learning approach that induced a new bilingual dictionary iteratively, achieving comparable results with only 25 word pairs. Conneau et al. [15] showed that they could build a high-quality bilingual dictionary without cross-lingual supervision. Their method leveraged both the domain-adversarial training approach and an iterative refinement procedure. Artetxe et al. [16] proposed a new unsupervised approach to learn cross-lingual embedding mappings by exploiting the structural similarity of the embeddings.

There are several studies on handling noise in parallel data. For example, Taghipour et al. [17] used a probability density estimation algorithm to detect outliers in parallel data. Cui et al. [18] proposed a graph-based random walk algorithm to compute the quality score of each sentence pair. Junczys-Dowmunt [19] introduced a dual conditional cross-entropy filtering, which computes cross-entropy scores based on the two translation models trained on clean data. These studies focused on filtering noise in the parallel data crawled from the web, instead of synthetic parallel data.

## 3. Proposed Method

### 3.1. Neural Machine Translation

A standard state-of-the-art NMT system follows the encoder-decoder framework. It includes two main components: an encoder network and a decoder network [20]. Given a source and target sentence pair $\left(X,Y\right)$, where $X=x_{1},\ldots ,x_{M}$ and $Y=y_{1},\ldots ,y_{N}$, the encoder network first takes source sentence *X* as an input and generates a list of fixed-size vectors $S=s_{1},\ldots ,s_{M}$, whose size is the length of the source sentence. Next, the decoder network predicts each token sequentially by maximizing the conditional probability:

$$p\left(y_{t}|y_{1},\ldots ,y_{t-1},S\right)=\mathrm{softmax}\left(W_{o}\;h_{t}\right),$$

where $W_{o}$ is the weight of the output softmax layer and $h_{t}$ is the target hidden state at time step *t*.

Given a parallel corpus *D*, the training objective is to minimize the cross-entropy loss:

$$L=\sum_{\left(X,Y\right)\in D}\;\sum_{t=1}^{N}\log\;p\left(y_{t}|y_{1},\ldots ,y_{t-1},S\right).$$

### 3.2. Back-Translation for NMT

Back-translation is a technique that employs target-language monolingual data in training the NMT system without changing its network architecture. Given a sentence-aligned parallel dataset $D_{p}={\left\{\left(X_{p}^{n},Y_{p}^{n}\right)\right\}}_{n=1}^{N}$ and a monolingual dataset in the target language $D_{tm}={\left\{Y_{tm}^{\left(m\right)}\right\}}_{m=1}^{M}$, the process of back-translation includes the following steps. First, a translation model in the reverse direction ${\mathrm{NMT}}_{Y\to X}$ is trained with the parallel dataset $D_{p}$. Second, with the translation model ${\mathrm{NMT}}_{Y\to X}$, the target-language monolingual dataset $D_{tm}$ is back-translated into the source-language translations $D_{st}={\left\{X_{st}^{\left(m\right)}\right\}}_{m=1}^{M}$, which is then paired with $D_{tm}$, making up a synthetic parallel dataset $D_{syn}={\left\{\left(X_{st}^{m},Y_{tm}^{m}\right)\right\}}_{m=1}^{M}$. Third, synthetic parallel dataset $D_{syn}$ and real parallel dataset $D_{p}$ are combined to train the main translation model ${\mathrm{NMT}}_{X\to Y}$.

### 3.3. Synthetic Parallel Data Filtering with Bilingual Word Embeddings

The filtering method introduced in this section is our main contribution. Our filtering method relies on cosine similarities of sentence embedding vectors in a common vector space. For each sentence *x*, we create its sentence embedding vector by accumulating word vectors $w_{1}$ to $w_{\left|x\right|}$, which are then averaged to form a single mean vector representation.

$$s_{x}=\frac{1}{\left|x\right|}\sum_{t=1}^{\left|x\right|}w_{t}$$

For each sentence pair $\left(x,y\right)$ in the synthetic parallel corpus, cosine similarity of $s_{x}$ and $s_{y}$ is computed as

$$\mathrm{similarity}\left(s_{x},s_{y}\right)=\frac{s_{x}\cdot s_{y}}{|s_{x}\left|\right|s_{y}|}\;.$$

Because the two sentences in each pair are written in different languages, it is necessary to ensure that the vector representations of these sentences are located in the same vector space.

A common approach to solve this problem is by using bilingual word embeddings. Following the work in [13,14,16], we first train word embeddings *X* and *Z* for the source and target language, respectively. Then, with a small bilingual dictionary, we learn a linear mapping *W* that minimizes the sum of squared Euclidean distances:

$$\underset{W}{\mathrm{argmin}}\sum_{i=1}^{n}{∥X_{i}\;W-Z_{i}∥}^{2},$$

where $X_{i}$ and $Z_{i}$ are the vector representations of word pairs in the bilingual dictionary.

Once the similarity scores of all sentence pairs are computed, we use a threshold value *t* to eliminate the sentence pairs with the scores below the threshold. The threshold value is computed by linearly scaling the similarity scores into the range of [0,1].

## 4. Experimental Setup

### 4.1. Datasets and Data Preprocessing

For Korean→English experiments, we used parallel training data released in IWSLT2017 [21] (the translation of TED talks). Besides, we used tst2016 and tst2017 as evaluation datasets (Available online: https://wit3.fbk.eu/). Monolingual data (English) for back-translation were obtained from the WMT2016 German–English news translation task. Dataset statistics is shown in Table 1.

The English sentences were tokenized and true-cased with Moses [22] preprocessing scripts. The Korean sentences were tokenized with Komoran (Available online: http://konlpy.org/en/) [23] tokenizer. We removed sentence pairs longer than 50 words and learned a joint source and target byte-pair encoding [24] with 32,000 merge operations.

All translation results reported in this paper were calculated in terms of single reference case-insensitive Bleu measured with Moses’ multi-bleu.perl script (Available online: https://github.com/moses-smt/mosesdecoder).

### 4.2. Models and Hyperparameters

The NMT system we used for evaluation is the OpenNMT [25] implementation of the Transformer [26] model. We followed the settings of the base model described in the paper, i.e., 6 attention blocks in the encoder and decoder, the embedding of size 512, and feed-forward dimension 2048. We used 8 attention heads, and we averaged the last 10 checkpoints, which were saved every 10,000 training steps.

The NMT system used for back-translation was an encoder–decoder model based on a 4-layer recurrent neural network (RNN). Specifically, we used the long short-term memory (LSTM) [27] and the attention mechanism proposed by Luong et al. [28]. We set hidden units to 1024, dropout rate to 0.2, and mini-batch size to 128. We trained the model with the stochastic gradient descent algorithm using a learning rate of 1.0, and we generally followed the learning rate decay scheme stated in [1].

The bilingual word embedding model used in our filtering method was obtained as follows. First, we trained word embeddings for Korean and English with fastText toolkit (Available online: https://fasttext.cc/) [29] on Wikipedia data (Available online: https://dumps.wikimedia.org/). Next, we created a list of English words by selecting the top 4500 most frequent words in the English Wikipedia data; function words and stop words were not included in the list. Subsequently, a bilingual (Korean and English) speaker translated all English words into Korean. Finally, we used existing approaches (Available online: https://github.com/artetxem/vecmap) to learn linear transformation matrix *W* with the word embeddings and the bilingual dictionary.

## 5. Experimental Results and Discussion

### 5.1. Quality of Bilingual Word Embeddings

To evaluate the quality of bilingual word embeddings, we created a word translation task that considered the translation accuracy of the given source words. The test set used in this task contains 500 word pairs that were uniformly selected from the bilingual dictionary. The bilingual word embeddings were obtained by applying existing approaches: Supervised [13], Identical [14], and Unsupervised [16]. These approaches mainly differ in which bilingual word pairs are used in learning linear transformation. Specifically, the Supervised method learns a mapping using all word pairs in a bilingual dictionary, the Identical method uses identical character strings as bilingual signal, and the Unsupervised method exploits the structural similarity of the embeddings instead of a bilingual dictionary.

Table 2 shows the quality of bilingual word embeddings in terms of word translation accuracy. As shown in Table 2, the supervised mapping method, trained with a bilingual dictionary of 4000 word pairs, achieved 42.60% accuracy, outperforming the other two approaches in our experiment. Therefore, we decided to choose the supervised method to build bilingual word embeddings in the following experiments.

### 5.2. Size of Synthetic Datasets

Sennrich [6] showed that the translation performance decreases if the size of synthetic data is too large compared to real data. Moreover, Fadaee and Monz [30] found that the model trained on 1:4 real-to-synthetic ratio of training data achieved slight improvements over the model trained on 1:1 training data. Because the size of real parallel data used in our experiments is relatively small, we explored various ratios of synthetic data to test which ratio achieves the best results.

Table 3 presents the translation performance of the systems trained on different ratios of the training data. The baseline model was trained on only real parallel data, whereas the “+ synthetic” models were trained on concatenated real and synthetic data. All models trained with additional synthetic data significantly outperformed the baseline model. In addition, models trained with synthetic data of ratio 1:5 outperformed the ratio 1:1 by a large margin. It is in line with the findings of Fadaee and Monz [30]. To our surprise, the 1:10 ratio of real-to-synthetic data performed best in our experiments. Hence, when the size of the real parallel corpus is relatively small, more synthetic data is required to obtain the best translation performance.

### 5.3. Quality of Filtered Synthetic Data

Subsequently, we analyze the quality of synthetic data filtered on two different approaches: “Sent-Bleu” and “Sent-BiEMB.” For this experiment, we sorted all the sentence pairs in the filtered synthetic data by their similarity scores. Next, we selected the top-ranked 200,000 and 400,000 sentence pairs and constructed new datasets: Top200k and Top400k. Afterward, we trained two NMT systems for each dataset and evaluated their performances on the test sets. The “Sent-Bleu” filtering method proposed by [7] removed noisy synthetic data based on sentence-level Bleu scores. The scores were calculated using the monolingual target sentences as a reference and synthetic target sentences as candidates. The synthetic target sentences were generated by translating source sentences in the synthetic parallel data into the target language. Here, the “Sent-BiEMB” is our proposed filtering method described in Section 3.3. In this experiment, the real parallel data were excluded.

As shown in Table 4, for Top200k synthetic data, the “Sent-BiEMB” model achieves 8.34 and 7.33 Bleu points, outperforming the “Sent-Bleu” by +2.64 and +2.04 Bleu points, on tst2016 and tst2017. Similar improvements are observed for Top400k synthetic data. The result indicates that our proposed method “Sent-BiEMB” is more effective than “Sent-Bleu” for filtering noise in synthetic data.

### 5.4. Performance of Proposed Method with a Combination of Real and Synthetic Data

In this section, we investigate the effects of different filtering methods on translation performance. The results are shown in Table 5. All models were trained on a concatenated real parallel data with filtered synthetic parallel data. The baseline was the best model trained on 1:10 real-to-synthetic ratio of training data described in Section 5.2.

As shown in Table 5, the filtering method based on sentence-level Bleu scores did not improve translation performance. This indicates that sentence-level Bleu is not as reliable as a filtering metric when the size of synthetic data is large. It is also in line with the result in [7].

Meanwhile, all “Sent-BiEMB” models outperformed the strong baseline model on both tst2016 and tst2017. The model with a similarity threshold of 0.3 achieved the best result, outperforming the baseline by +0.72 and +0.62 Bleu points (We have performed a test of significance on improvements of the proposed model over the baseline. The test statics (z-score) of tst2016 and tst2017 are 12.63 and 14.05, respectively. The P-value of both test sets is less than 0.0001. Therefore, we conclude that the gains over the baseline are statistically significant). It confirms that filtering noisy sentence pairs from synthetic parallel data with bilingual word embeddings improves the translation models.

## 6. Conclusions

In this paper, we proposed a simple approach to filtering noisy sentence pairs from a synthetic parallel corpus generated with back-translation. We measured the sentence-level similarities between the synthetic source and the monolingual target sentence by using bilingual word embeddings. The distributed representation of words was also considered in the proposed method. We observed gains in translation performance by removing noisy sentence pairs with the proposed method.

In future research, we plan to further analyze the types of noise in the synthetic parallel data generated by back-translation and investigate their effects on translation performance. Additionally, we will evaluate our filtering method on other language pairs.

## Figures and Tables

**Table 1:** Statistics for the Korean–English translation datasets.

Dataset	Source	Target	Sentences
IWSLT2017	Korean	English	207 K
WMT2016	-	English	4.5 M
tst2016	Korean	English	1024
tst2017	Korean	English	1036

**Table 2:** Word translation accuracy of bilingual word embeddings on Korean→English word translation task.

Bilingual Mappings	Accuracy
Supervised	42.60%
Identical	41.58%
Unsupervised	40.16%

**Table 3:** Korean→English translation performance (Bleu) on IWSLT test sets (TED talks) with different ratios of *real:syn* data.

Model	Size	tst2016	tst2017
Baseline	207 K	14.46	12.84
+ synthetic (1:1)	414 K	15.55	13.67
+ synthetic (1:5)	1.2 M	17.44	15.24
+ synthetic (1:10)	2.2 M	**18.01**	**15.48**
+ synthetic (1:20)	4.2 M	16.26	14.03

**Table 4:** Quality of filtered synthetic data in terms of translation performance of (Bleu) on IWSLT test set. Systems are trained using only synthetic parallel data filtered with Sent-Bleu and Sent-BiEMB.

Model	Synthetic Data	tst2016	tst2017
Sent-Bleu	Top200k	5.70	5.27
Sent-BiEMB	Top200k	**8.34 (+2.64)**	**7.33 (+2.06)**
Sent-Bleu	Top400k	8.41	7.38
Sent-BiEMB	Top400k	**10.05 (+1.64)**	**9.03 (+1.65)**

**Table 5:** Korean→English translation performance of (Bleu) on IWSLT test sets (TED talks). Systems differ in how the synthetic parallel data is filtered.

Model	Threshold	tst2016	tst2017
Baseline	None	18.01	15.48
Sent-Bleu	0.1	17.97	15.66
Sent-Bleu	0.2	17.67	15.26
Sent-Bleu	0.3	17.45	15.39
Sent-Bleu	0.4	16.93	14.65
Sent-BiEMB	0.1	18.03	15.73
Sent-BiEMB	0.2	18.11	15.70
Sent-BiEMB	0.3	**18.73 (+0.72)**	**16.10 (+0.62)**
Sent-BiEMB	0.4	18.20	15.97
